# Supplementary material for: Crosstalk among Alternative Polyadenylation, Genetic Variants and Ubiquitin Modification Contribute to Lung Adenocarcinoma Risk
Source: Int J Mol Sci. 2024 Jul 24;25(15):8084. doi: 10.3390/ijms25158084 (PMC11311407; doi:10.3390/ijms25158084)
Supplement: Supplementary file 1 [file ijms-25-08084-s001.zip › ijms-3070188-supplementary.pdf]

Supplementary Table S1. Detail information of 49 cis apaQTL-SNPs

No.	SNP	Gene	CHR	Position(hg37)	Minor allele	Major allele	P [a]	MAF(CHB)
1	rs10497760	*HECW2*	2	195444739	C	T	0.985	0.282
2	rs56336904	*TRIM24*	7	138046555	C	T	0.185	0.451
3	rs9649632	*TRIM24*	7	138073334	G	T	0.286	0.466
4	rs73450170	*TRIM24*	7	138088756	T	C	0.483	0.102
5	rs10245726	*TRIM24*	7	138089192	G	T	0.447	0.112
6	rs6952043	*TRIM24*	7	138093064	G	A	0.684	0.107
7	rs4732314	*TRIM24*	7	138095400	T	G	0.488	0.306
8	rs10248876	*TRIM24*	7	138104511	T	C	0.649	0.442
9	rs6951998	*TRIM24*	7	138106707	C	T	0.546	0.364
10	rs6467782	*TRIM24*	7	138244045	T	C	0.106	0.437
11	rs4581063	*PDLIM2*	8	23116392	G	A	0.217	0.189
12	rs7216737	*RNF213*	17	77848006	T	C	0.185	0.078
13	rs112649406	*RNF213*	17	78277425	A	G	0.268	0.146
14	rs7502437	*RNF213*	17	78282148	C	T	0.139	0.393
15	rs74790219	*RNF213*	17	78292779	G	A	0.424	0.238
16	rs6565666	*RNF213*	17	78292945	A	G	0.487	0.175
17	rs7222014	*RNF213*	17	78293469	A	G	0.993	0.286
18	rs6565667	*RNF213*	17	78294175	C	A	0.256	0.451
19	rs4889845	*RNF213*	17	78303560	G	A	0.302	0.461
20	rs58124977	*RNF213*	17	78305444	C	G	0.381	0.383
21	rs8074015	*RNF213*	17	78306280	G	A	0.567	0.447
22	rs4890012	*RNF213*	17	78319380	C	G	0.505	0.417
23	rs12051723	*RNF213*	17	78324259	T	G	0.392	0.272
24	rs7216493	*RNF213*	17	78327358	A	G	0.354	0.461
25	rs4889846	*RNF213*	17	78329624	G	A	0.561	0.408
26	rs12150357	*RNF213*	17	78339009	T	C	0.324	0.345
27	rs34907722	*RNF213*	17	78339618	G	T	0.58	0.15
28	rs35096485	*RNF213*	17	78374969	A	G	0.584	0.461
29	rs58958230	*RNF213*	17	78375336	T	G	0.201	0.417
30	rs9893968	*RNF213*	17	78390195	A	G	0.158	0.456
31	rs8076103	*RNF213*	17	78390646	G	C	0.194	0.437
32	rs41299816	*RNF213*	17	78397756	T	C	0.919	0.136
33	rs8065290	*RNF213*	17	78401588	T	G	0.06	0.364
34	rs8081162	*RNF213*	17	78404709	A	G	0.988	0.141
35	rs41301932	*RNF213*	17	78406974	A	G	0.002	0.053
36	rs4494603	*RNF213*	17	78408213	G	C	0.001	0.068
37	rs35832855	*RNF213*	17	78410749	C	T	0.763	0.209
38	rs34357871	*RNF213*	17	78414824	A	G	0.661	0.175
39	rs12938834	*RNF213*	17	78415668	T	G	0.929	0.481
40	rs34369758	*RNF213*	17	78416218	G	A	0.486	0.15
41	rs9890400	*RNF213*	17	78416493	G	A	0.023	0.422

42	rs12946823	*RNF213*	17	78416793	G	T	0.08	0.393
43	rs9914610	*RNF213*	17	78417220	A	G	0.351	0.364
44	rs56066320	*RNF213*	17	78418906	G	C	0.001	0.073
45	rs9906819	*RNF213*	17	78426075	C	A	0.253	0.417
46	rs13341123	*RNF213*	17	78427033	T	G	0.003	0.063
47	rs2725391	*RNF213*	17	79192430	T	C	0.811	0.398
48	rs1048775	*RNF213*	17	79202329	C	G	0.443	0.286
49	rs2659007	*RNF213*	17	79217478	A	G	0.223	0.233

[a] Logistic regression analysis adjusted for age in FLCCA GWAS.

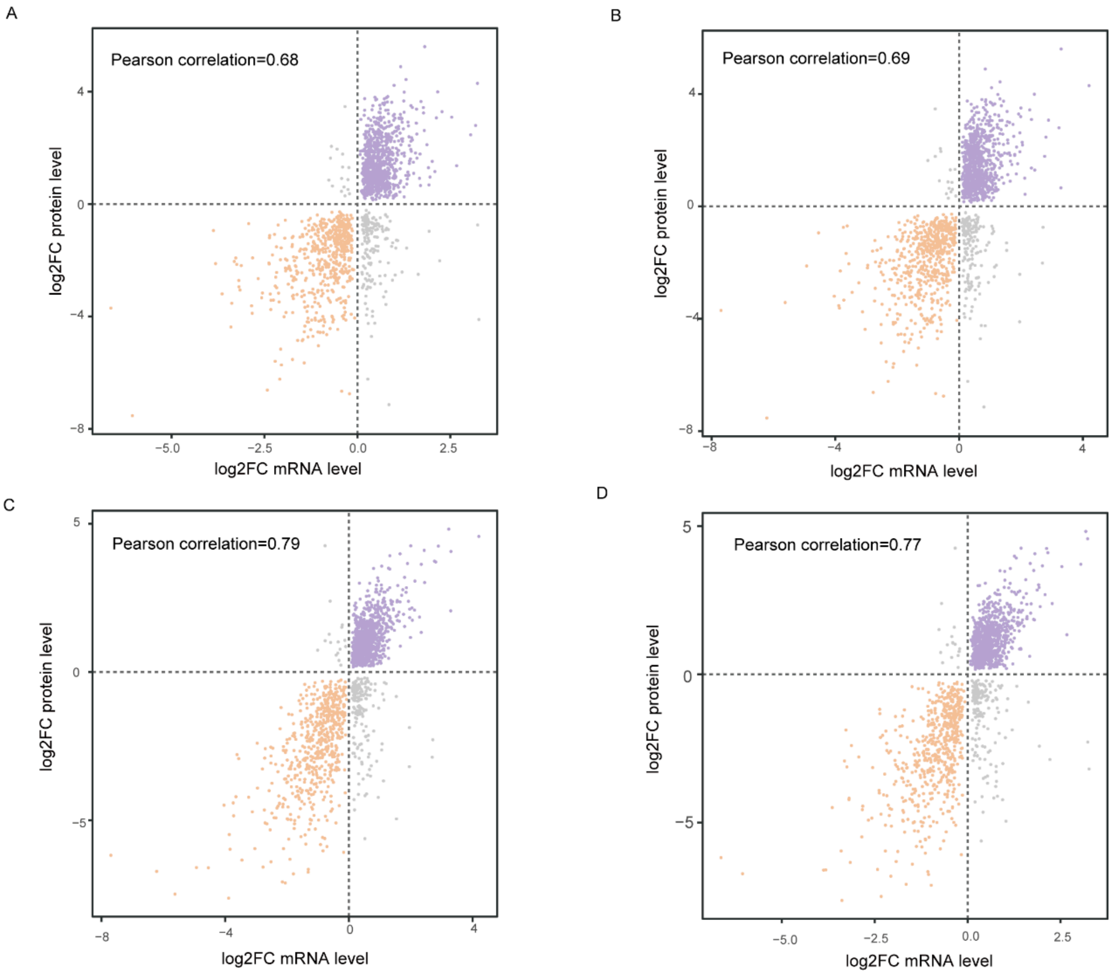

Supplementary Figure S1. Quadrant plot of consistency and discrepancy in protein and mRNA Expression. (A) Chinese LUAD protein expression and Chinese LUAD gene expression (correlation: 0.68); (B) Chinese LUAD protein expression and TCGA-LUAD gene expression (correlation: 0.69); (C)CPTAC LUAD protein expression and TCGA LUAD gene expression (correlation: 0.79); (D) CPTAC LUAD protein expression and Chinese LUAD gene expression (correlation: 0.77).

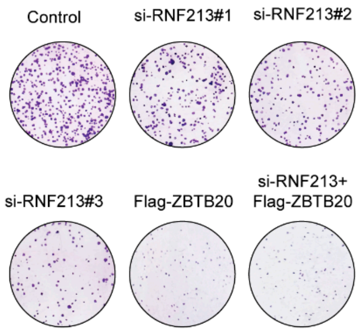

Supplementary Figure S2. The representative images of Colony formation of SPCA1 cells in indicated groups.
